# The Beekeeping Sector of Kazakhstan and Contributions to the Pharmaceutical Industry: Promising Flavonoids with the Pharmacokinetic Properties Via the Computational Evaluations

**DOI:** 10.12688/f1000research.188625.2

**Published:** 2026-09-28

**Authors:** Şeyda Kaya, Zeliha Selamoglu, Sevgi Durna Daştan, Tulkinzhon Gaipov, Taner Daştan, Nurdana Salybekova

**Affiliations:** 1 Department of Medical Services and Techniques, Medical Laboratory Techniques Program, Vocational School of Health Services, Gaziantep Islam Science and Technology University, Gaziantep, Turkey; 2 Department of Medical Biology, Medicine Faculty, Niğde Omer Halisdemir University, Niğde, Turkey; 3 Department of Biology, Faculty of Sciences, Khoja Akhmet Yassawi International Kazakh-Turkish University, Turkestan, Kazakhstan; 4 Department of Biology, Faculty of Sciences, Western Caspian University, Baku, Azerbaijan; 5 Department of Biology, Faculty of Science, Sivas Cumhuriyet University, Sivas, Turkey; 6 Beekeeping Development, Application and Research Center, Sivas Cumhuriyet University, Sivas, Turkey; 7 Center for Sustainable Development and Scientific Research, Khoja Akhmet Yassawi International Kazakh-Turkish University, Turkistan, Kazakhstan; 8 Department of Biochemistry, Faculty of Science, Sivas Cumhuriyet University, Sivas, Turkey

**Keywords:** ADME, Bee products, Beekeeping, Drug-likeness, Flavonoids, Kazakhstan, Sustainable development

## Abstract

**Background:**

Bee products are rich sources of bioactive flavonoids with potential applications in nutraceutical and pharmaceutical research. However, differences in the physicochemical and pharmacokinetic properties of individual flavonoids may substantially influence their drug-likeness and bioavailability. This study aimed to characterize the pharmacokinetic, physicochemical, and drug-likeness profiles of four representative flavonoids commonly associated with bee products myricetin, galangin, kaempferol, and quercetin.

**Methods:**

*In silico* pharmacokinetic and drug-likeness analyses were performed using the SwissADME web platform. The investigated compounds were evaluated in terms of physicochemical properties, lipophilicity, aqueous solubility, gastrointestinal absorption, blood-brain barrier (BBB) permeability, P-glycoprotein (P-gp) substrate status, and compliance with established drug-likeness rules, including Lipinski, Veber, Egan, and Muegge criteria.

**Results:**

All four flavonoids exhibited favorable molecular properties and an identical predicted bioavailability score of 0.55. Galangin, kaempferol, and quercetin showed high predicted gastrointestinal absorption and fully complied with the Lipinski, Veber, Egan, and Muegge drug-likeness criteria. In contrast, myricetin exhibited comparatively lower predicted gastrointestinal absorption, which may be associated with its higher polarity, greater hydrogen-bonding capacity, and elevated topological polar surface area. None of the investigated flavonoids was predicted to penetrate the BBB or to act as a P-gp substrate. Variations in hydroxyl substitution were associated with marked differences in lipophilicity, aqueous solubility, membrane permeability, and overall pharmacokinetic behavior. Among the compounds evaluated, galangin displayed the most balanced physicochemical and medicinal chemistry profile, whereas kaempferol and quercetin also demonstrated favorable characteristics for oral drug development.

**Conclusions:**

The findings indicate that flavonoids associated with Kazakhstan bee products possess distinct but generally favorable drug-likeness and predicted pharmacokinetic profiles. In particular, galangin emerged as the most balanced candidate among the compounds examined. Integrating *in silico* pharmacokinetic assessment with knowledge of bee-product composition may contribute to the scientific evaluation, quality standardization, and value-added utilization of Kazakhstan’s apicultural resources, while providing a basis for further experimental investigation of their nutraceutical and pharmaceutical potential.

## 1. Introduction

Honey bees ( *Apis mellifera* L.) play an indispensable role in ecosystem sustainability and agricultural productivity through pollination while simultaneously producing a wide variety of biologically valuable hive products. Owing to its vast geographical area, remarkable ecological heterogeneity, and climatic diversity extending from alpine mountains and temperate forests to steppe and semi-arid ecosystems, Kazakhstan represents one of the most promising apicultural regions in Central Asia. Beekeeping is present in different geographical parts of Kazakhstan, from the central steppes to southern highlands, and this study is an effort to contribute to the ongoing national effort for the development of Southern Kazakhstan, which has a particular standing with its suitable climatic zones and floral microhabitats. Beekeeping in the districts of Southern Kazakhstan serves a dual purpose, both for commercial production and for providing materials for research efforts to characterization of biochemical compositions and developing streamlined production frameworks. Notably, these beekeeping facilities have also provided material for laboratory-based chemical characterization efforts. These diverse environments provide abundant nectar- and pollen-producing plants that substantially influence the phytochemical composition and biological quality of locally produced honey, pollen, propolis, royal jelly, and other bee products. Particularly, the Turkestan, Almaty, East Kazakhstan, and Zhetysu regions possess rich floral resources and relatively low levels of industrial pollution, creating favorable conditions for sustainable beekeeping and the production of high-quality bee products suitable for domestic consumption and international markets ( Bradbear, 2009; Dimeyeva et al., 2016; Gritsenko et al., 2023).

Beyond their traditional nutritional role, bee products are now widely recognized as functional foods, nutraceuticals, and promising sources of pharmaceutical lead compounds. Increasing scientific evidence demonstrates that honey, pollen, propolis, bee bread, royal jelly, and bee venom exhibit antioxidant, antimicrobial, anti-inflammatory, immunomodulatory, antidiabetic, neuroprotective, cardioprotective, and anticancer activities. These biological properties are largely attributed to their complex phytochemical composition, particularly phenolic acids and flavonoids, which have become major targets of natural product research and pharmaceutical development ( Selamoglu et al., 2025a; Selamoglu et al., 2025b).

Among the numerous phytochemicals identified in bee products, flavonoids represent one of the most biologically active groups because of their ability to modulate oxidative stress, inflammation, apoptosis, and metabolic signaling pathways. Their abundance is strongly influenced by botanical origin, geographical location, climatic conditions, and seasonal variation. Kazakhstan’s exceptional botanical diversity therefore provides a valuable source of chemically diverse bee products containing numerous pharmacologically important flavonoids. Representative floral resources and their associated flavonoids reported in Kazakhstan bee products are summarized in Table 1. Compounds such as myricetin, quercetin, galangin, chrysin, pinocembrin, kaempferol, and rutin have consistently been identified in honey, pollen, and propolis originating from temperate floral sources. Collectively, these molecules contribute to the antioxidant capacity and therapeutic potential of bee products while representing attractive candidates for future nutraceutical and pharmaceutical development.

**Table 1.** Representative floral resources and major flavonoids associated with bee products from different regions of Kazakhstan.

Region	Predominant floral resources	Representative flavonoids frequently reported in bee products	Potential biological relevance
Turkestan	*Medicago sativa* (Alfalfa)	Myricetin, Quercetin	Antioxidant, antidiabetic, anti-inflammatory
Almaty	*Fagopyrum esculentum* (Buckwheat)	Quercetin, Rutin	Cardioprotective, antioxidant
East Kazakhstan	Wild forest flora, Poplar species	Galangin, Chrysin, Pinocembrin	Antimicrobial, neuroprotective
Zhetysu	Multifloral vegetation	Kaempferol, Quercetin	Antioxidant, metabolic regulation

* Representative flavonoids compiled from published studies describing botanical sources commonly associated with bee products in Kazakhstan and neighboring temperate regions.

Among these flavonoids, myricetin, galangin, quercetin, kaempferol have generally emerged as the most promising bioactive compounds owing to their broad spectrum of pharmacological activities. Numerous experimental studies have demonstrated that these chemicals possess potent antioxidant, anti-inflammatory, antidiabetic, neuroprotective, cardioprotective, antimicrobial, and anticancer properties through modulation of multiple molecular targets associated with oxidative stress, glucose metabolism, mitochondrial function, inflammatory signaling, and programmed cell death. Furthermore, they have also been shown to regulate pathways implicated in diabetes mellitus, cardiovascular diseases, neurodegenerative disorders, chronic inflammatory diseases, and several types of cancer. Despite these promising biological activities, the successful pharmaceutical development remains constrained by uncertainties regarding their pharmacokinetic behavior, gastrointestinal absorption, membrane permeability, metabolic stability, and oral bioavailability. Therefore, evaluating these characteristics have become an essential prerequisite for assessing its translational potential as a natural therapeutic agent.

Recent advances in computational pharmacology have transformed early-stage natural product research by enabling rapid prediction of physicochemical properties, pharmacokinetic behavior, and drug-likeness before experimental validation. Among the available computational platforms, SwissADME has become one of the most widely accepted tools for predicting molecular descriptors, gastrointestinal absorption, blood-brain barrier permeability, cytochrome P450 interactions, medicinal chemistry parameters, and compliance with established drug-likeness rules. Such *in silico* approaches considerably reduce development time and experimental costs while facilitating the prioritization of promising natural compounds for subsequent biological investigation. Although the biological activities of these compounds have been extensively investigated, relatively few studies have examined their pharmacokinetic characteristics and drug-likeness specifically within the context of bee products from Kazakhstan, despite the country’s remarkable apicultural biodiversity and increasing importance in the international honey market. Scientific characterization of bioactive constituents from Kazakhstan bee products is becoming increasingly important not only for understanding their pharmaceutical potential but also for supporting quality standardization, authentication, traceability, value-added commercialization, and sustainable development of the national beekeeping sector. As illustrated in Figure 1, Kazakhstan’s diverse floral resources support the production of chemically rich bee products that may serve as valuable reservoirs of pharmacologically relevant flavonoids. Therefore, the present study aimed to comprehensively evaluate the physicochemical properties, pharmacokinetic profile, medicinal chemistry characteristics, and drug-likeness of some bioactive molecules using the SwissADME platform, thereby providing a scientific basis for future pharmaceutical exploitation of Kazakhstan bee products and contributing to the development of sustainable, value-added apicultural products.

**Figure 1.**
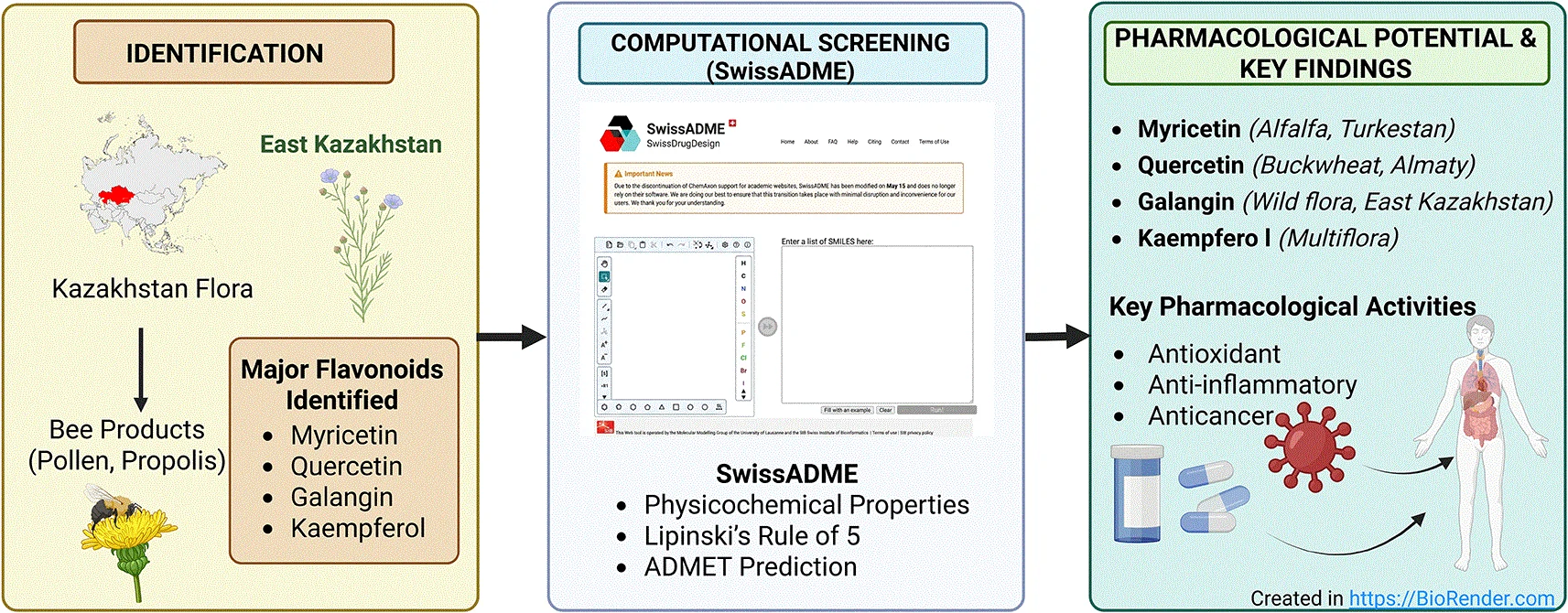
Kazakhstan’s diverse floral resources support the honey bee products.

## 2. Material and method

The PubChem database was used for the querying and obtaining SMILES code for the myricetin, galangin, quercetin, kaempferol molecules ( Kim et al., 2025). Following the addition of SMILES code, the SwissADME web-based tool ( www.swissadme.ch) was used for analyses. The SwissADME as a tool provides predictions for diverse parameters that include molecular physicochemistry, pharmacokinetics, similarity indices, chemical compatibility, and was developed and designed by the Swiss Institute of Bioinformatics (SIB). The ADMET results and BOILED-Egg graph results were shared in the results section ( Daina and Zoete, 2016; Daina et al., 2017). TPSA is calculated using the fragment-based topological method; the Consensus Log Po/w is the arithmetic mean of five independent prediction models (XLOGP3, WLOGP, MLOGP, SILICOS-IT, and iLOGP); the BOILED-Egg model classifies gastrointestinal absorption and blood-brain barrier permeation using WLOGP and TPSA within a Monte-Carlo-optimized probability ellipse; and the bioavailability score follows the rule-based classification originally described by ( Martin, 2005).

## 3. Results

East Kazakhstan, Pavlodar, Abai, Almaty, Turkestan, and Zhetysu are some of the major geographical areas where Kazakhstan’s honey production is centered. With 603.7 tonnes of honey sent overall in 2024, Uzbekistan emerged as the top export destination, making up 73.2% of the entire export volume. Canada (8.9%), China (6.1%), and Saudi Arabia (3.8%) were secondary export destinations; sales to the US and Russia were still quite small. Honey imports, on the other hand, totaled 1,657.3 tons, suggesting a significant trade imbalance. 97.9% of all honey imports came from Russia, underscoring Kazakhstan’s heavy reliance on one outside supplier. Kazakhstan’s honey trade’s import and export structure in 2024 ( Table 2).

**Table 2.** Structure of Kazakhstan’s honey exports and imports in 2024.

Trade flow	Partner country	Volume (t)	Share (%)
**Exports**	Uzbekistan	442.0	73.2
	Canada	54.0	8.9
	China	36.8	6.1
	Saudi Arabia	23.0	3.8
	Russia	21.1	3.5
	United States	18.5	3.1
	Other countries	8.3	1.4
**Imports**	Russia	1,621.9	97.9
	Germany	21.0	1.3
	China	7.1	0.4
	Other countries	7.3	0.4

The molecular structure of myricetin, a natural flavonoid, is shown in Figure 2, and its general chemical properties are listed in Table 3. To assess whether the molecule is a suitable chemical molecule in terms of bioavailability, the SwissADME Bioavailability Radar graph is shown in Figure 2 ( Daina et al., 2017). This graph allows for the rapid visualization of the drug candidate molecule to be used. According to this, the myricetin molecule has ideal ranges for parameters indicating its fat solubility (LIPO), size (SIZE) dependent on molecular weight, insolubility (INSOLU) indicating whether the molecule dissolves in water, and flexibility (FLEX) indicating the molecule’s ability to change conformation based on the number of rotatable bonds. The molecule’s polarity (POLAR) and aromatic/double bond content (INSATU) ratio being high indicates that it is outside the ideal range ( Figure 2). Similar to myricetin, the bioavailability radar of galangin demonstrated favorable values for lipophilicity, molecular size, flexibility, and solubility. In contrast to myricetin, galangin exhibited a lower polarity owing to its reduced number of hydroxyl groups, positioning most physicochemical descriptors within the optimal range for oral bioavailability. These findings suggest a more balanced physicochemical profile that may support improved membrane permeability. Kaempferol also displayed a favorable bioavailability profile characterized by appropriate lipophilicity, molecular size, and flexibility. Although its polarity remained moderately elevated because of multiple hydroxyl substituents, the deviation from the optimal range was less pronounced than that observed for myricetin. Overall, the radar plot indicated physicochemical characteristics compatible with acceptable drug-likeness. The bioavailability radar of quercetin revealed physicochemical properties largely comparable to those of kaempferol. While molecular size, lipophilicity, flexibility, and solubility fell within the desirable range, increased polarity associated with the presence of multiple hydroxyl groups remained the principal factor limiting its overall bioavailability profile. Nevertheless, quercetin retained favorable drug-likeness characteristics according to the SwissADME assessment ( Figure 2).

**Figure 2.**
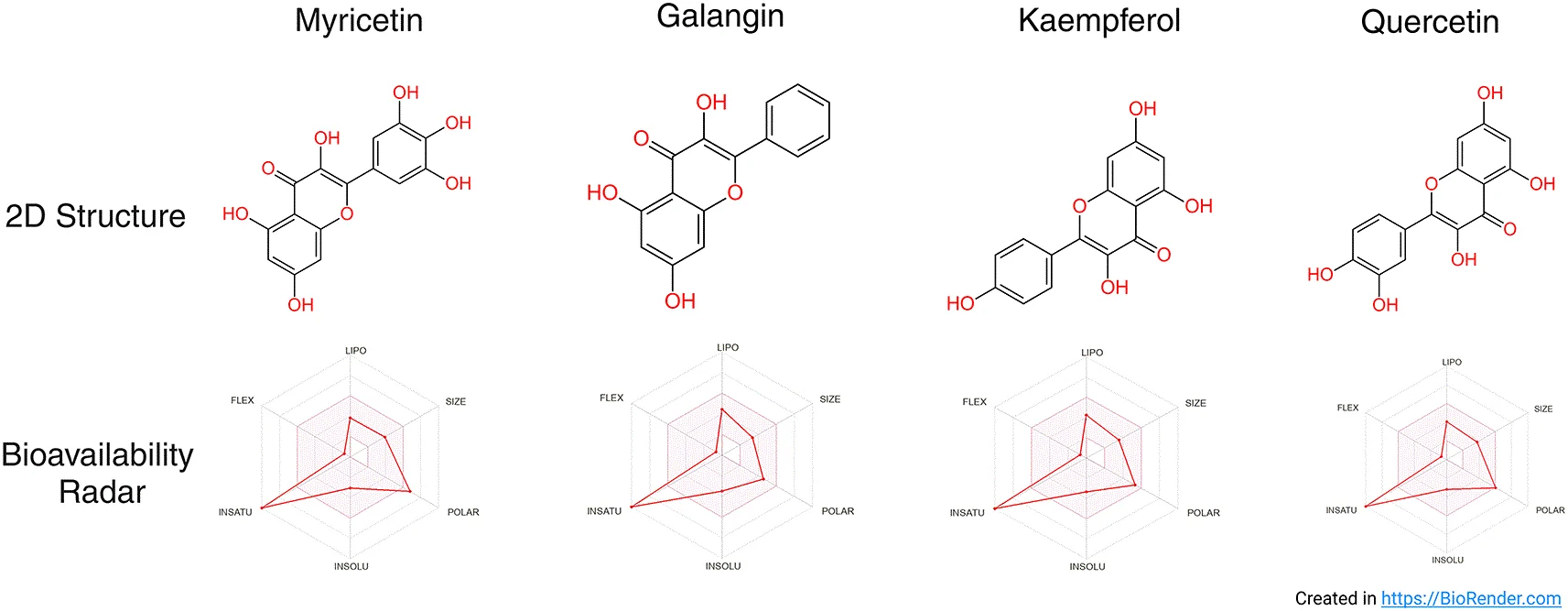
Myricetin, galangin, kaempferol, and quercetin molecule and Bioavailability Radar chart.

**Table 3.** The properties of myricetin, galangin, kaempferol, and quercetin. See Figure 2 for the corresponding 2D chemical structures.

Property	Myricetin	Galangin	Kaempferol	Quercetin
**PubChem ID**	5281672	5281616	5280863	5280343
**IUPAC Name**	3,5,7-trihydroxy-2-(3,4,5-trihydroxyphenyl)chromen-4-one	3,5,7-trihydroxy-2-phenylchromen-4-one	3,5,7-trihydroxy-2-(4-hydroxyphenyl)chromen-4-one	2-(3,4-dihydroxyphenyl)-3,5,7-trihydroxychromen-4-one
**Canonical SMILES**	C1 = C(C=C(C(=C1O)O)O)C2 = C(C(=O)C3 = C(C=C(C=C3O2)O)O)O	C1 = CC=C(C=C1)C2 = C(C(=O)C3 = C(C=C(C=C3O2)O)O)O	C1 = CC(=CC=C1C2 = C(C(=O)C3 = C(C=C(C=C3O2)O)O)O)O	C1 = CC(=C(C=C1C2 = C(C(=O)C3 = C(C=C(C=C3O2)O)O)O)O)O

The fundamental physicochemical properties of the molecule are listed in Table 4. According to the properties listed here, the myricetin molecule has a molecular weight of 318.24 g/mol and a TPSA (Topological Polar Surface Area) of 151.59 Å ^2^. According to Lipinski’s ‘Five Rules’, a molecular weight of <500 g/mol is ideally considered for a molecule to be drug-like. ( Lipinski et al., 2001). According to Hydrogen Bond Capacity, the myricetin molecule has 6 Donor and 8 Acceptor points. This number is very high. The molecule bonds very tightly with water, which makes it difficult to pass through fatty tissues (cell membranes). The principal differences among the flavonoids were observed in their hydrogen-bonding capacity and molecular polarity. Galangin contained the fewest hydrogen-bond acceptors (5) and donors (3), whereas myricetin exhibited the highest values (8 acceptors and 6 donors), reflecting its extensive hydroxylation pattern. Kaempferol and quercetin displayed intermediate hydrogen-bonding capacities, with six and seven acceptors and four and five donors, respectively. These structural differences were also reflected in the topological polar surface area (TPSA). Galangin showed the lowest TPSA (90.90 Å ^2^), followed by kaempferol (111.13 Å ^2^) and quercetin (131.36 Å ^2^), whereas myricetin exhibited the highest polarity with a TPSA of 151.59 Å ^2^. The gradual increase in TPSA closely paralleled the increasing number of hydroxyl substituents, suggesting that hydrogen-bonding capacity is the primary determinant of polarity among the investigated flavonoids. Since elevated TPSA generally reduces passive membrane permeability, galangin is expected to possess more favorable permeability characteristics than the more highly hydroxylated flavonoids, particularly myricetin. Overall, the results indicate that although the investigated flavonoids share a common structural scaffold, variations in hydroxyl substitution substantially influence their physicochemical profiles and may consequently affect their pharmacokinetic behavior.

**Table 4.** The physicochemical properties of myricetin, galangin, kaempferol, and quercetin.

Property	Myricetin	Galangin	Kaempferol	Quercetin
**Formula**	C _15_H _10_O _8_	C _15_H _10_O _5_	C _15_H _10_O _6_	C _15_H _10_O _7_
**Molecular weight**	318.24 g/mol	270.24 g/mol	286.24 g/mol	302.24 g/mol
**Num. heavy atoms**	23	20	21	22
**Num. arom. Heavy atoms**	16	16	16	16
**Fraction Csp3**	0.00	0.00	0.00	0.00
**Num. rotatable bonds**	1	1	1	1
**Num. H-bond acceptors**	8	5	6	7
**Num. H-bond donors**	6	3	4	5
**Molar Refractivity**	80.06	73.99	76.01	78.03
**TPSA (Å** ^**2**^ **)**	151.59	90.90	111.13	131.36

Considering the lipophilicity and water solubility values of myricetin given in Table 5, the Consensus Log P _o/w_ is determined as 0.38, is determined as 0.38, the lowest value among the four flavonoids. This indicates that myricetin is comparatively hydrophilic, which is consistent with its reduced predicted membrane permeability. The lipophilicity profiles of the investigated flavonoids were generally comparable, although galangin exhibited the highest lipophilic character because of its lower hydroxyl substitution. Conversely, myricetin displayed the lowest consensus Log P value, reflecting its higher polarity. Kaempferol and quercetin showed intermediate lipophilicity, suggesting a balance between aqueous solubility and membrane permeability. Despite sharing a common flavonoid backbone, the four compounds exhibited distinct lipophilicity values that closely reflected their degree of hydroxyl substitution. Galangin showed the highest consensus Log P value (1.63), indicating the greatest lipophilic character among the investigated flavonoids, whereas myricetin displayed the lowest value (0.38), consistent with its extensive hydroxylation and increased polarity. Kaempferol (1.20) and quercetin (0.79) exhibited intermediate lipophilicity, representing a gradual transition between galangin and myricetin. The same structural trend was reflected in the predicted water solubility. According to the ESOL model, all four flavonoids were classified as soluble, although quantitative solubility differed among compounds. Myricetin demonstrated the highest predicted aqueous solubility (2.92 × 10 ^−1^ mg/mL), followed by quercetin (1.96 × 10 ^−1^ mg/mL), kaempferol (1.32 × 10 ^−1^ mg/mL), and galangin (8.74 × 10 ^−2^ mg/mL). Comparable trends were observed using the Ali and SILICOS-IT prediction models, confirming that increasing hydroxyl substitution generally enhanced water solubility while reducing lipophilicity. Overall, the physicochemical predictions revealed an inverse relationship between lipophilicity and aqueous solubility across the investigated flavonoids. Galangin exhibited the highest lipophilicity but the lowest predicted aqueous solubility, whereas myricetin displayed the opposite profile. Kaempferol and quercetin demonstrated intermediate characteristics, suggesting a more balanced distribution between hydrophilic and lipophilic properties that may influence their pharmacokinetic behavior. Taken together, these results indicate a consistent structure–property relationship among the four investigated flavonoids. The number of hydroxyl substituents on the flavonoid backbone followed the order galangin < kaempferol < quercetin < myricetin, and this ranking closely paralleled the observed trends in TPSA, hydrogen-bond donor/acceptor count, and predicted aqueous solubility, all of which increased progressively in the same order. Conversely, Consensus Log Po/w, skin permeability (Log Kp), and predicted gastrointestinal absorption decreased progressively along this same series. This inverse relationship between hydroxylation degree and lipophilicity/permeability suggests that hydroxyl substitution is the principal structural determinant of the pharmacokinetic differences observed among these flavonoids, consistent with the general medicinal chemistry principle that increased hydrogen-bonding capacity reduces passive membrane permeability.

**Table 5.** Lipophilicity and water solubility of myricetin, galangin, kaempferol, and quercetin.

Property	Myricetin	Galangin	Kaempferol	Quercetin
**Lipophilicity**				
**Log Po/w (iLOGP)**	Temporarily unavailable	Temporarily unavailable	Temporarily unavailable	Temporarily unavailable
**Log Po/w (XLOGP3)**	1.23	2.30	1.94	1.59
**Log Po/w (WLOGP)**	1.69	2.58	2.28	1.99
**Log Po/w (MLOGP)**	−1.08	0.52	−0.03	−0.56
**Log Po/w (SILICOS-IT)**	−0.34	1.11	0.62	0.14
**Consensus Log Po/w**	0.38	1.63	1.20	0.79
**Water Solubility**				
**Log S (ESOL)**	−3.04	−3.49	−3.33	−3.19
**Solubility (ESOL)**	2.92e-01 mg/ml; 9.19e-04 mol/l	8.74e-02 mg/ml; 3.23e-04 mol/l	1.32e-01 mg/ml; 4.63e-04 mol/l	1.96e-01 mg/ml; 6.49e-04 mol/l
**Class (ESOL)**	Soluble	Soluble	Soluble	Soluble
**Log S (Ali)**	−4.01	−3.85	−3.90	−3.96
**Solubility (Ali)**	3.10e-02 mg/ml; 9.75e-05 mol/l	3.85e-02 mg/ml; 1.42e-04 mol/l	3.62e-02 mg/ml; 1.26e-04 mol/l	3.32e-02 mg/ml; 1.10e-04 mol/l
**Class (Ali)**	Moderately soluble	Soluble	Soluble	Soluble
**Log S (SILICOS-IT)**	−2.19	−3.94	−3.36	−2.77
**Solubility (SILICOS-IT)**	2.05e+00 mg/ml; 6.43e-03 mol/l	3.12e-02 mg/ml; 1.16e-04 mol/l	1.26e-01 mg/ml; 4.40e-04 mol/l	5.07e-01 mg/ml; 1.68e-03 mol/l
**Class (SILICOS-IT)**	Soluble	Soluble	Soluble	Soluble

Tables 6 and 7 shows that myricetin has low gastrointestinal absorption (GI absorption) and is not permeant to the blood-brain barrier (BBB). According to the data in the table generated by SwissADME predictions and the BOILED-Egg graph results, myricetin shows low gastrointestinal absorption. The BOILED-Egg (Brain or IntestinaL EstimateD permeation) model visualizes a molecule’s Gastrointestinal absorption (HIA) and Blood-Brain Barrier (BBB) passage. The X-axis provides information about the lipophilicity (WLOGP) of the molecule, while the Y-axis provides information about the polarity (TPSA) of the molecule ( Tables 6 and 7). As indicated in Figure 3, myricetin exhibits low gastrointestinal absorption and no blood-brain barrier permeability ( Daina and Zoete, 2016). Galangin exhibited the most favorable oral pharmacokinetic profile, with high predicted gastrointestinal absorption and compliance with the major drug-likeness rules. Kaempferol and quercetin also demonstrated acceptable pharmacokinetic characteristics, whereas myricetin showed comparatively lower gastrointestinal absorption, primarily attributable to its high polarity and elevated TPSA. None of the four compounds was predicted to readily cross the blood-brain barrier, indicating limited central nervous system exposure.

**Table 6.** Pharmacokinetics of myricetin, galangin, kaempferol, and quercetin.

Property	Myricetin	Galangin	Kaempferol	Quercetin
**GI absorption**	Low	High	High	High
**BBB permeant**	No	No	No	No
**P-gp substrate**	No	No	No	No
**CYP1A2 inhibitor**	Temporarily unavailable	Temporarily unavailable	Temporarily unavailable	Temporarily unavailable
**CYP2C19 inhibitor**	Temporarily unavailable	Temporarily unavailable	Temporarily unavailable	Temporarily unavailable
**CYP2C9 inhibitor**	Temporarily unavailable	Temporarily unavailable	Temporarily unavailable	Temporarily unavailable
**CYP2D6 inhibitor**	Temporarily unavailable	Temporarily unavailable	Temporarily unavailable	Temporarily unavailable
**CYP3A4 inhibitor**	Temporarily unavailable	Temporarily unavailable	Temporarily unavailable	Temporarily unavailable
**Log Kp (skin permeation, cm/s)**	−7.37	−6.32	−6.67	−7.01

**Table 7.** Druglikeness and medicinal chemistry of myricetin, galangin, kaempferol, and quercetin.

Property	Myricetin	Galangin	Kaempferol	Quercetin
**Druglikeness**				
**Lipinski**	Yes; 1 violation (NHorOH>5)	Yes; 0 violation	Yes; 0 violation	Yes; 0 violation
**Ghose**	Yes	Yes	Yes	Yes
**Veber**	No; 1 violation (TPSA>140)	Yes	Yes	Yes
**Egan**	No; 1 violation (TPSA>131.6)	Yes	Yes	Yes
**Muegge**	No; 2 violations (TPSA>150, H-don>5)	Yes	Yes	Yes
**Bioavailability Score**	0.55	0.55	0.55	0.55
**Medicinal Chemistry**				
**PAINS**	1 alert: catechol_A	0 alert	0 alert	1 alert: catechol_A
**Brenk**	1 alert: catechol	0 alert	0 alert	1 alert: catechol
**Leadlikeness**	Yes	Yes	Yes	Yes
**Synthetic accessibility**	3.27	3.12	3.14	3.23

**Figure 3.**
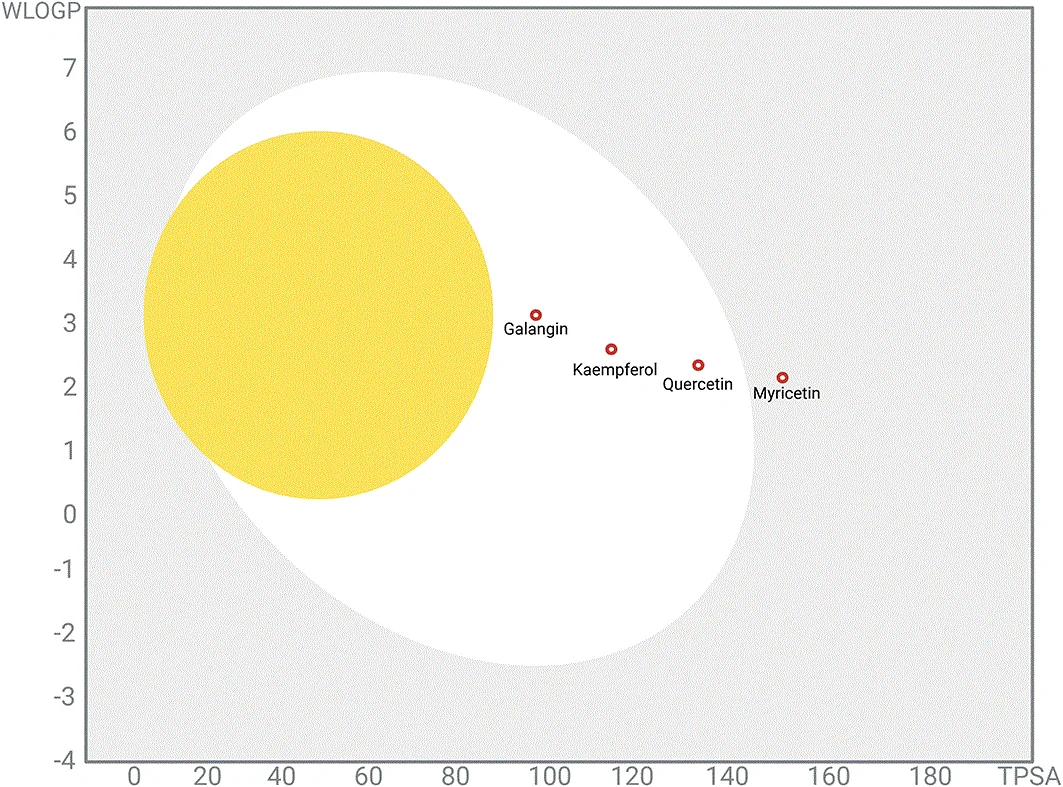
BOILED-Egg result of all molecules.

Marked differences were observed in gastrointestinal absorption despite the structural similarity of the four compounds. Myricetin was predicted to exhibit low gastrointestinal (GI) absorption, whereas galangin, kaempferol, and quercetin all showed high predicted GI absorption. This difference is consistent with the higher polarity and larger topological polar surface area of myricetin compared with the other flavonoids. None of the investigated compounds was predicted to penetrate the blood-brain barrier (BBB), suggesting limited central nervous system exposure following systemic administration. Likewise, none of the flavonoids was identified as a substrate of P-glycoprotein (P-gp), indicating that active P-gp-mediated efflux is unlikely to represent a major limitation to their intestinal disposition. Predicted skin permeability also differed slightly among the compounds. Galangin exhibited the highest skin permeability (Log Kp = −6.32 cm/s), followed by kaempferol (−6.67 cm/s) and quercetin (−7.01 cm/s), whereas myricetin showed the lowest predicted skin permeability (−7.37 cm/s). These results closely paralleled the lipophilicity profiles of the flavonoids, with the less polar compounds demonstrating greater membrane permeability than the highly hydroxylated myricetin. Overall, the pharmacokinetic predictions indicate a progressive improvement in membrane permeability from myricetin to galangin, reflecting the influence of hydroxyl substitution on molecular polarity and suggesting that relatively less hydroxylated flavonoids may possess more favorable absorption characteristics.

The predicted drug-likeness and medicinal chemistry properties of myricetin, galangin, kaempferol, and quercetin are summarized in Table 7. All four flavonoids satisfied the Ghose criteria and were classified as lead-like compounds with an identical predicted bioavailability score of 0.55, indicating comparable potential for oral bioavailability. In addition, all compounds exhibited relatively low synthetic accessibility scores (3.12–3.27), suggesting moderate feasibility for chemical synthesis. Among the investigated flavonoids, galangin, kaempferol, and quercetin demonstrated the most favorable drug-likeness profiles. These compounds fully complied with Lipinski’s rule of five as well as the Veber, Egan, and Muegge filters, indicating physicochemical characteristics generally associated with orally active drug candidates ( Veber et al., 2002). In contrast, myricetin exhibited one violation of Lipinski’s rule due to its high number of hydrogen-bond donors (NH or OH > 5), together with additional violations of the Veber, Egan, and Muegge criteria resulting from its elevated topological polar surface area (TPSA) and extensive hydroxylation. Medicinal chemistry assessment further distinguished the investigated flavonoids. Galangin and kaempferol showed no PAINS or Brenk structural alerts, suggesting a lower probability of assay interference and fewer structural liabilities during early-stage drug discovery. Conversely, both myricetin and quercetin generated one PAINS alert (catechol_A) and one Brenk alert (catechol), reflecting the presence of catechol moieties that may contribute to nonspecific biological responses in high-throughput screening assays. Overall, the predicted drug-likeness profiles indicate that galangin and kaempferol possess the most favorable balance between physicochemical properties and medicinal chemistry parameters, whereas the extensive hydroxylation of myricetin results in several rule-based violations despite maintaining an acceptable bioavailability score and lead-likeness classification.

The BOILED-Egg model further illustrated the differences in predicted gastrointestinal absorption among the investigated flavonoids. Galangin occupied the region associated with favorable passive intestinal absorption, whereas kaempferol and quercetin were positioned close to the optimal absorption zone. In contrast, myricetin remained outside the optimal gastrointestinal absorption region because of its higher polarity. None of the investigated compounds was predicted to efficiently penetrate the blood-brain barrier.

CYP450 inhibition data were temporarily unavailable for all four compounds at the time of analysis ( Table 6), precluding any conclusions regarding cytochrome P450-mediated drug–drug interaction potential. This represents a limitation of the current SwissADME-based assessment and warrants re-evaluation once platform predictions become available, or verification using an alternative *in silico* CYP inhibition model. The Log Kp (skin permeation) value of −7.37 cm/s again indicates that myricetin has a low capacity to pass through the skin, suggesting that its application to the skin or mucous membranes is inefficient. Lipinski’s 5 Rules are an important parameter for drug design, evaluating the drug potential of an orally administered compound. According to these rules, myricetin’s Hydrogen Bond Donors (H-Bond Donors) should be less than 5, whereas NHorOH >5 is a rule violation. According to Veber’s rules, a TPSA value greater than 140 Å ^2^ for myricetin is also considered a violation ( Veber et al., 2002). The bioavailability score for myricetin was found to be 0.55. This score indicates that the molecule has a 55% probability of exhibiting bioavailability greater than 10%. This is a standard value for a plant compound. PAINS (Pan-Assay Interference Compounds) is used to screen for chemical groups that give misleading results in high-throughput screening of molecules with drug potential. The catechol A group motifs in myricetin are listed as PAINS alerts ( Baell and Holloway, 2010). The value indicating the ease of synthesizing myricetin in the laboratory is stated as 3.27, which is close to 1, indicating moderate ease ( Ertl and Schuffenhauer, 2009). This is usually a assay artifact arising from its chemical structure rather than a true therapeutic effect. Therefore, biological studies conducted with this molecule should be interpreted with great caution. Therefore, rather than attempting to use the myricetin molecule as a systemic drug in the form of a pill that mixes with the blood and spreads throughout the body, it may be more reasonable and logical to use it topically, applied to the skin surface, without conducting detailed biological activity experiments or working on animal models gastrointestinal inflammatory conditions such as IBD, myricetin may act locally via direct mucosal contact rather than through systemic absorption.

## 4. Discussion

With its ecological diversity, geographical features, and with the existence of floral microhabitats, Kazakhstan is one of the suitable countries for sustainable beekeeping practices. Beekeeping is not limited to obtaining quality honey, it is equally about to obtain other beekeeping products such as pollen, propolis, royal jelly, bee bread, and bee venom, and the biological activities and potencies of such products are largely dependent on how these products are obtained. Sustainable practices require optimal conditions both for collection and storage.

### 4.1 Kazakhstan as a source of high-value bee products

Kazakhstan possesses one of the largest natural landscapes in Central Asia, encompassing extensive steppe ecosystems, mountainous regions, forest habitats, and semi-arid environments that collectively provide exceptional floral diversity for apiculture. This ecological heterogeneity directly influences the botanical origin and phytochemical composition of bee products, resulting in considerable variability in their biological and pharmaceutical properties ( Herrera Cerquera and Betancurt, 2025). Unlike many intensively cultivated agricultural regions, large areas of Kazakhstan remain relatively free from industrial pollution, thereby supporting the production of bee products with high natural quality and reduced environmental contamination. Consequently, Kazakhstan has emerged as an important producer of honey, pollen, propolis, royal jelly, and other hive products that are increasingly attracting scientific and commercial interest ( Bradbear, 2009; Dimeyeva et al., 2016; Gritsenko et al., 2023).

The medicinal value of bee products is largely attributed to their rich composition of phenolic acids, flavonoids, terpenoids, vitamins, enzymes, and other secondary metabolites. Among these constituents, flavonoids have received particular attention because of their multifunctional biological activities, including antioxidant, anti-inflammatory, antimicrobial, antidiabetic, cardioprotective, and neuroprotective effects ( Selamoglu et al., 2025a; Selamoglu et al., 2025b). Since the phytochemical composition of bee products strongly depends on floral origin, geographical location, climatic conditions, and seasonal variations, characterization of region-specific bioactive compounds has become essential not only for understanding biological activity but also for establishing quality standards, geographical authentication, and value-added commercialization. From this perspective, Kazakhstan represents an underexplored yet highly promising reservoir of naturally occurring bioactive molecules with potential applications extending far beyond traditional food products.

### 4.2 Comparative pharmacokinetic characteristics of major flavonoids identified in kazakhstan bee products

Phenolic compounds have been identified in Kazakhstani monofloral honeys and may contribute to their observed antioxidant and enzyme-inhibitory activities ( Ongalbek et al., 2026). Their biological activities extend beyond simple antioxidant functions and include modulation of inflammatory signaling, glucose metabolism, mitochondrial homeostasis, apoptosis, autophagy, and numerous intracellular pathways associated with chronic diseases. Because these compounds frequently coexist within honey, pollen, propolis, and other bee products, evaluating their pharmacokinetic behavior collectively provides a more comprehensive understanding of the therapeutic potential of Kazakhstan apicultural products than examining a single constituent alone.

The present study therefore investigated representative flavonoids commonly associated with Kazakhstan bee products, including myricetin, quercetin, galangin, kaempferol, and other structurally related compounds. Although these flavonoids share the common flavone or flavonol backbone, their pharmacokinetic profiles differ considerably because of variations in hydroxyl substitution patterns, molecular polarity, hydrogen-bonding capacity, and lipophilicity. Such structural differences substantially influence gastrointestinal absorption, membrane permeability, blood-brain barrier penetration, metabolic stability, and ultimately their suitability as pharmaceutical candidates.

Among the investigated compounds, myricetin exhibited one of the highest molecular polarities owing to its multiple hydroxyl groups. While this structural feature is advantageous for radical scavenging and antioxidant activity, it simultaneously reduces passive diffusion across biological membranes, resulting in relatively low predicted gastrointestinal absorption and limited oral bioavailability. Similar observations have been consistently reported in previous computational and experimental studies, highlighting the intrinsic trade-off between antioxidant potency and pharmacokinetic performance commonly observed among highly hydroxylated flavonoids. Consequently, despite its remarkable biological activities, myricetin may require advanced formulation strategies such as nanoencapsulation, phospholipid complexes, or prodrug approaches to maximize its therapeutic efficacy ( Imran et al., 2021; Yang et al., 2020). Due to such features, myricetin may act as an enzyme inhibitor, epigenetic regulator, and signalling modulator relevant to neurodegenerative processes. In particular, myricetin has been shown to inhibit lipopolysaccharide-induced neuroinflammation, supporting its potential relevance as a neuroprotective agent ( Jang et al., 2020). From the point of epigenetic regulation myricetin is found to be interacting with KDM4A–C (particularly KDM4B) molecule, which is a histone lysine demethylase responsible for removing methyl groups from lysine residues on histones, hence highlighting its possible activities in cancer pathogenesis, cell-cycle regulation, and cellular stress responses ( Liu et al., 2022). Myricetin has also demonstrated a protective effect in a murine model of dextran sulphate sodium-induced ulcerative colitis, suggesting potential relevance to inflammatory bowel disease (IBD) ( Zhao et al., 2013). There are supporting claims where myricetin containing natural products contributing positively to immune modulation and to alleviation of oxidative stress that leads to various pathologies including cardiovascular and neurodegenerative diseases ( Wang et al., 2019; Huang et al., 2024; Wang et al., 2010).

On the other hand, quercetin, another abundant flavonol frequently identified in bee products, demonstrated a more balanced physicochemical profile. Previous investigations have shown that quercetin combines strong antioxidant and anti-inflammatory properties with relatively improved membrane permeability compared with myricetin, primarily because of its lower hydroxylation degree. Numerous studies have further demonstrated that quercetin modulates insulin signaling, inflammatory cytokines, endothelial dysfunction, and oxidative stress, supporting its potential application in metabolic and cardiovascular diseases. Nevertheless, quercetin also suffers from limited aqueous solubility and rapid metabolism, factors that continue to stimulate the development of novel delivery systems aimed at improving its clinical bioavailability ( Table 8).

**Table 8.** Comparative evaluation of biopharmaceutic potential.

Compound	Drug-likeness	ADME	General profile
Galangin	⭐⭐⭐⭐⭐	⭐⭐⭐⭐⭐	Best
Kaempferol	⭐⭐⭐⭐⭐	⭐⭐⭐⭐⭐	Best
Quercetin	⭐⭐⭐⭐⭐	⭐⭐⭐⭐	Good
Myricetin	⭐⭐⭐	⭐⭐⭐	Biologically potent

The present evaluation also highlights the pharmaceutical relevance of galangin, a flavonol that possesses fewer hydroxyl groups and consequently exhibits greater lipophilicity than both myricetin and quercetin. Increased lipophilicity generally favors membrane permeability and may contribute to improved intestinal absorption. Galangin has attracted increasing attention because of its antimicrobial, anti-inflammatory, anticancer, and neuroprotective properties, while several experimental studies suggest its ability to regulate oxidative stress and apoptosis through multiple signaling pathways. The comparatively favorable physicochemical characteristics observed for galangin indicate that it may represent an attractive scaffold for medicinal chemistry optimization and future drug development ( Table 8).

Similarly, kaempferol has emerged as another important flavonol naturally occurring in pollen, honey, and propolis. Extensive pharmacological investigations have associated kaempferol with antioxidant, antidiabetic, cardioprotective, neuroprotective, and anticancer activities. From a pharmacokinetic perspective, kaempferol generally exhibits intermediate characteristics between quercetin and galangin, combining acceptable membrane permeability with relatively favorable drug-likeness properties. Such characteristics may partially explain the increasing interest in kaempferol as a lead compound for metabolic and inflammatory disorders ( Table 8).

In conclusion, the therapeutic properties of the four bioactive compounds are presented comparatively in Table 8. Collectively, the comparative evaluation performed in this study demonstrates that no single flavonoid possesses an ideal pharmacokinetic profile. Instead, each compound exhibits distinct advantages and limitations arising from its chemical structure. Highly hydroxylated molecules generally provide superior antioxidant capacity but reduced membrane permeability, whereas less polar flavonoids often display improved pharmacokinetic characteristics with comparatively lower radical-scavenging activity. This balance between biological potency and pharmacokinetic behavior represents one of the central challenges in natural-product-based drug discovery.

Importantly, bee products should not be viewed as sources of isolated compounds alone. Rather, they represent complex phytochemical matrices in which multiple flavonoids coexist and may exert additive or synergistic biological effects. Growing evidence suggests that combinations of flavonoids can modulate multiple molecular targets simultaneously, potentially producing greater therapeutic efficacy than individual purified constituents. Therefore, evaluating the collective pharmacokinetic characteristics of representative bee-product flavonoids provides a more realistic representation of their pharmaceutical potential than focusing exclusively on a single molecule.

Overall, the SwissADME predictions presented in this study provide a valuable preliminary framework for prioritizing flavonoids according to their drug-likeness and pharmacokinetic properties. Although computational analyses cannot replace experimental pharmacokinetic investigations, they offer an efficient strategy for identifying the most promising candidates for subsequent *in vitro*, *in vivo*, and clinical validation. Consequently, the present findings support the view that Kazakhstan bee products constitute a rich source of structurally diverse flavonoids with complementary pharmacological characteristics and considerable potential for future nutraceutical and pharmaceutical applications.

## 5. Conclusions

Beyond the pharmacokinetic characterization of kaempferol, galangin, myricetin, and quercetin the present study highlights the strategic importance of Kazakhstan’s beekeeping sector as a sustainable source of bioactive compounds with pharmaceutical potential. Integrating computational pharmacology with apicultural research provides a valuable framework for identifying high-value natural products, supporting evidence-based apitherapeutic applications, and enhancing the international competitiveness of Kazakhstan’s bee products through scientific validation. It should be stressed that while *in silico* analyses generally lead to promising prospects for various natural bioactive substances, experimental studies combining both *in vitro* and *in vivo* analyses should be carried out to validate computational results, which is the case for myricetin investigated in this paper. While bee products have an increased interest in academic research, vigorous studies remain as necessity for precise results, which is also necessary to further understand and validate its promising clinical potential as a natural product. Such research would also contribute to improvement of beekeeping practices. As a direct follow-up to the present computational screening, future work will focus on the experimental validation of the predicted pharmacokinetic differences among the investigated flavonoids through *in vitro* permeability assays, alongside antioxidant and anti-inflammatory bioassays using extracts derived from Kazakhstan-sourced bee products.

## Ethics statement

This study is entirely computational ( *in silico*), no human, animal, or biological data were used, and ethical approval was therefore not required.

## Declaration of AI use

AI-based tools were used for initial drafting and language editing of the manuscript. All scientific decisions and interpretations were made exclusively by the authors.

## Data Availability

Underlying data, Zenodo: SwissADME-based ADME/pharmacokinetic prediction data for myricetin, quercetin, kaempferol, and galangin. https://doi.org/10.5281/zenodo.21858254 ( Kaya et al., 2026). Data are available under the terms of the Creative Commons Attribution 4.0 International license (CC-BY 4.0). No extended data associated with this article.
