# Supplementary material for: The Beekeeping Sector of Kazakhstan and Contributions to the Pharmaceutical Industry: Promising Flavonoids with the Pharmacokinetic Properties Via the Computational Evaluations
Source: F1000Res. 2026 Sep 28;15:1422. Originally published 2026 Aug 25. [Version 2] doi: 10.12688/f1000research.188625.2 (PMC13583342; doi:10.12688/f1000research.188625.2)
Supplement: Peer review history

# Reviewer report: Reviewer response for version 1

**DOI:** 10.5256/f1000research.208184.r518703

**Published:** 2026-09-16

**Authors:** Ramin Ekhteiari Salmas

**Affiliations:** 1 King's College London, London, England, UK

This study performs an *in silico* computational analysis using SwissADME to evaluate the physicochemical, pharmacokinetic, and drug-likeness profiles of four major flavonoids. Galangin, kaempferol, and quercetin demonstrated high predicted gastrointestinal absorption and full compliance with major drug-likeness rules. In contrast, myricetin showed lower predicted GI absorption and several rule violations due to its high polarity and extensive hydroxylation. None of the compounds crossed the blood-brain barrier or acted as P-glycoprotein substrates.

 From my side, the manuscript can be accepted after addressing the following revisions:

1. Please include the 2D chemical structures of the molecules listed in Table 3.
2. Did you observe any meaningful relationships between the chemical structures and their physicochemical properties? If so, please discuss them in the text.
3. Keep only the PubChem IDs and 2D structures for the representative molecules in Table 3. Move the remaining to the Supporting Information.
4. Please briefly discuss the underlying methodology SwissADME uses to calculate these results.
5. Are there plans for future experimental studies to validate these analyses?

Is the work clearly and accurately presented and does it cite the current literature?

Yes

If applicable, is the statistical analysis and its interpretation appropriate?

Yes

Are all the source data underlying the results available to ensure full reproducibility?

Yes

Is the study design appropriate and is the work technically sound?

Yes

Are the conclusions drawn adequately supported by the results?

Yes

Are sufficient details of methods and analysis provided to allow replication by others?

Yes

Reviewer Expertise:

My field is drug discovery, studying small molecules to understand their potential roles.

I confirm that I have read this submission and believe that I have an appropriate level of expertise to confirm that it is of an acceptable scientific standard.

# Response

**Published:** 2026-09-17

**Authors:** Zeliha Selamoglu

**Affiliations:** Nigde Ömer Halisdemir University, Turkey

We sincerely thank the reviewer for the positive and constructive evaluation of our manuscript, and for confirming that the study design, methodology, data availability, and conclusions meet an acceptable scientific standard. We have addressed each of the reviewer's five suggestions below, and believe these revisions have improved the clarity and completeness of the manuscript.

1. We thank the reviewer for this suggestion. We would like to clarify that the 2D chemical structures of all four flavonoids are already presented in Figure 2, alongside their corresponding SwissADME bioavailability radar plots, so that structural features and predicted physicochemical behavior can be visually compared side by side. We had intentionally kept Table 3 limited to textual identifiers (PubChem CID, IUPAC name, canonical SMILES) to avoid duplicating the same graphical content in two places within the manuscript. To address the reviewer's concern for ease of reference, we have added an explicit cross-reference in the Table 3 caption directing readers to Figure 2 for the corresponding 2D structures.
2. Yes. Our results reveal a clear structure–property relationship among the four flavonoids: increasing hydroxyl substitution (galangin < kaempferol < quercetin < myricetin) is associated with a progressive increase in TPSA, hydrogen-bond donor/acceptor count, and predicted aqueous solubility, alongside a progressive decrease in Consensus Log Po/w, skin permeability, and predicted gastrointestinal absorption. We thank the reviewer for prompting us to make this relationship explicit, as it had previously been discussed only in a fragmented manner across separate paragraphs. A new synthesizing paragraph has been added to the Results section summarizing this structure–property trend.
3. We thank the reviewer for this suggestion. However, we would prefer to retain the IUPAC names and canonical SMILES strings within Table 3 in the main text, as these identifiers are essential for reproducibility and independent verification of the compounds studied, and their removal to the Supporting Information could reduce accessibility for readers directly comparing structural and pharmacokinetic data. As noted in our response to Comment 1, we have added a cross-reference in the Table 3 caption directing readers to Figure 2 for the corresponding 2D structures, which we believe addresses the reviewer's core concern regarding visual clarity without requiring readers to consult supplementary material for basic chemical identifiers.
4. We thank the reviewer for highlighting this gap. A short paragraph has been added to the Materials and Methods section summarizing the computational basis of the key predictions used: TPSA is calculated using the fragment-based topological method; the Consensus Log Po/w is the arithmetic mean of five independent prediction models (XLOGP3, WLOGP, MLOGP, SILICOS-IT, and iLOGP); the BOILED-Egg model classifies gastrointestinal absorption and blood-brain barrier permeation using WLOGP and TPSA within a Monte-Carlo-optimized probability ellipse; and the bioavailability score follows the rule-based classification originally described by (Martin et al. 2005).
5. Yes. As a direct follow-up to this *in silico* screening, we plan to pursue experimental validation of the predicted pharmacokinetic differences among the four flavonoids, particularly the comparatively lower gastrointestinal absorption of myricetin, using *in vitro* permeability assays. In parallel, we intend to characterize the biological activity of these compounds as they occur naturally in Kazakhstan-sourced bee products (honey, pollen, and propolis), including antioxidant and anti-inflammatory bioassays, to bridge the present computational findings with experimentally verifiable outcomes. A statement to this effect has been added to the Conclusion.

We are grateful to the reviewer for their time, expertise, and valuable suggestions, which have helped us improve the manuscript. We hope that the revised version now meets the requirements for acceptance.

# Reviewer report: Reviewer response for version 1

**DOI:** 10.5256/f1000research.208184.r518694

**Published:** 2026-09-10

**Authors:** Kamala Badalova

**Affiliations:** 1 Azerbaijan Medical University, Baku, Baku, Azerbaijan

This article provides a concise in silico evaluation of four flavonoids—myricetin, galangin, kaempferol, and quercetin—associated with bee products, focusing on their physicochemical, pharmacokinetic, and drug-likeness properties. Using SwissADME, the study examines important parameters such as gastrointestinal absorption, lipophilicity, solubility, BBB permeability, P-glycoprotein activity, and compliance with established drug-likeness rules.

 A major strength of the study is its comparative approach, which clearly demonstrates how differences in hydroxyl substitution influence the properties of structurally related flavonoids. The finding that galangin has the most balanced overall profile is particularly interesting, while myricetin’s lower predicted gastrointestinal absorption highlights the potential impact of increased polarity and hydrogen-bonding capacity on bioavailability. The article is relevant to pharmacognosy, nutraceutical research, and the valorization of Kazakhstan’s apicultural resources. It offers useful preliminary evidence for prioritizing flavonoids—particularly galangin—for further experimental investigation, but additional laboratory validation is necessary before claims about therapeutic or drug-development potential can be established.

Is the work clearly and accurately presented and does it cite the current literature?

Yes

If applicable, is the statistical analysis and its interpretation appropriate?

Yes

Are all the source data underlying the results available to ensure full reproducibility?

Yes

Is the study design appropriate and is the work technically sound?

Yes

Are the conclusions drawn adequately supported by the results?

Yes

Are sufficient details of methods and analysis provided to allow replication by others?

Yes

Reviewer Expertise:

Medicinal chemistry

I confirm that I have read this submission and believe that I have an appropriate level of expertise to confirm that it is of an acceptable scientific standard.

# Response

**Published:** 2026-09-17

**Authors:** Zeliha Selamoglu

**Affiliations:** Nigde Ömer Halisdemir University, Turkey

We sincerely thank the reviewer for their thoughtful and encouraging evaluation of our manuscript. We are pleased that the reviewer found the comparative structure–property approach informative, and we agree that the identification of galangin as the most balanced candidate, alongside the reduced predicted bioavailability of myricetin, represents one of the more actionable findings of this study for future research prioritization. We also fully concur with the reviewer's note that experimental ( *in vitro/in vivo*) validation remains a necessary next step before any therapeutic or drug-development claims can be substantiated; we have been careful throughout the manuscript to frame our findings as computational predictions requiring further validation, and we appreciate the reviewer's reinforcement of this point. We are grateful for the reviewer's time and expertise in assessing this work.
